# Screening for Elevated Blood Lead Levels in Children: Assessment of Criteria and a Proposal for New Ones in France

**DOI:** 10.3390/ijerph121214989

**Published:** 2015-12-03

**Authors:** Anne Etchevers, Philippe Glorennec, Yann Le Strat, Camille Lecoffre, Philippe Bretin, Alain Le Tertre

**Affiliations:** 1 INSERM (National Institute of Health and Medical Research) U1085, Irset-Environmental and Occupational Health Research Institute, Rennes 35043, France; anne.etchevers@gmail.com; 2 EHESP (School of Public Health), Sorbonne Paris Cité, Rennes 35043, France; 3 InVS—French Institute for Public Health Surveillance, Saint Maurice 94415, France; y.lestrat@invs.sante.fr (Y.L.S.); c.lecoffre@invs.sante.fr (C.L.); a.letertre@invs.sante.fr (A.L.T.); 4 Ministry of Health, Directorate for Health, Paris 75350, France; Philippe.BRETIN@sante.gouv.fr

**Keywords:** lead poisoning, exposure, health, child, screening evaluation

## Abstract

The decline in children’s Blood Lead Levels (BLL) raises questions about the ability of current lead poisoning screening criteria to identify those children most exposed. The objectives of the study were to evaluate the performance of current screening criteria in identifying children with blood lead levels higher than 50 µg/L in France, and to propose new criteria. Data from a national French survey, conducted among 3831 children aged 6 months to 6 years in 2008–2009 were used. The sensitivity and specificity of the current criteria in predicting blood lead levels higher than or equal to 50 µg/L were evaluated. Two predictive models of BLL above 44 µg/L (for lack of sufficient sample size at 50 µg/L) were built: the first using current criteria, and the second using newly identified risk factors. For each model, performance was studied by calculating the area under the ROC (Receiver Operating Characteristic) curve. The sensitivity of current criteria for detecting BLL higher than or equal to 50 µg/L was 0.51 (0.26; 0.75) and specificity was 0.66 (0.62; 0.70). The new model included the following criteria: foreign child newly arrived in France, mother born abroad, consumption of tap water in the presence of lead pipes, pre-1949 housing, period of construction of housing unknown, presence of peeling paint, parental smoking at home, occupancy rates for housing and child’s address in a cadastral municipality or census block comprising more than 6% of housing that is potentially unfit and built pre-1949. The area under the ROC curve was 0.86 for the new model, *versus* 0.76 for the current one. The lead poisoning screening criteria should be updated. The risk of industrial, occupational and hobby-related exposure could not be assessed in this study, but should be kept as screening criteria.

## 1. Introduction

Children’s lead exposure has dramatically decreased in France as well as in many developing countries [1,2,3,4,5,6] over the past 15 years. The geometric mean Blood Lead Levels (BLL) was 14.9 μg/L (95% confidence interval (CI) = (14.5–15.4)) in children aged between 6 months and 6 years living in France in 2009 [7]. The prevalence of BLLs exceeding 100 µg/L (the current level of intervention in France) declined from 2.1% in 1995 to 0.1% in 2009 in 1 to 6 year-old children [7]. However many recent studies have shown that neurological and behavioral effects can occur at BLLs below 50 µg/L [4,8,9]. The French High Council for Public Health (HCSP) therefore recommended decreasing the current level of intervention from 100 to 50 μg/L [10]. As a consequence, the lead poisoning screening programs must now detect the 75,600 children expected to be above this new level [10]. The current national screening program is based on individual detection of lead risk factors during the medical check-ups at 9 and 24 months through an individual “lead risk” questionnaire [11,12]. The questionnaire includes eight self-reported criteria: child living in or frequently visiting a house built pre-1949 with peeling or chipping paint, child living in a house built pre-1949 with recent renovation or remodeling, child living with someone who has suffered from lead poisoning, child living with an adult whose job or hobby involves exposure to lead, child tending to scrape off and nibble paint, child having recently moved to France, child living or frequenting places near an industry emitting lead and child exposed to lead water risk [12]. A blood test is prescribed if the parents answer “yes” to one or more questions. In addition to individual screening, local data such as housing construction year and characteristics, corrosiveness of tap water, suspected use of lead-based paint in housing, cases of lead poisoning and census of industrial sites have been used occasionally or routinely to define screening areas. Despite many isolated initiatives, there is still a need for simple, standardized tools to help each French department locally [13].

As yet, no study has evaluated the performance of the current questionnaire in identifying children having BLLs ≥ 50 μg/L. The national survey of blood lead levels carried out in 2008–2009, known as Saturn-Inf [7], provided an opportunity to assess the predictive validity of current criteria for BLLs ≥ 50 μg/L. In this study, information on children’s lead exposure was reported by parents in similar conditions to those of the screening questionnaire. It was also an opportunity to test a better combination of criteria.

Moreover, given the decrease of BLLs and the widespread geographic distribution of lead exposure in housing at both national and local levels [14,15,16,17], the questionnaires should be administered based on other “high-risk population” targeting methods [13]. Previous studies have demonstrated the ability of geographical indicators of poverty and old housing to detect children at increased risk of elevated BLLs [18,19,20]. Using an ecological indicator based on a child’s census block and socioeconomic characteristics can be more predictive of elevated BLLs than the lead risk self-report questionnaire, as described by Kaplowitz *et al.* [21].

The first objective of this study was to assess the performance of the current lead risk screening questionnaire (defined to detect BLLs ≥ 100 µg/L) in detecting children with BLLs higher than 50 µg/L, the new intervention level. The second objective was to propose new criteria that would better detect children over this new intervention level. In particular we propose testing a geographic criterion that could help state health departments and physicians identify high-risk populations.

## 2. Methods

### 2.1. Study Population and Data Collection

Saturn-Inf (the national study of BLL) was carried out between September 2008 and April 2009 [7]. This cross-sectional survey involved 3831 children, aged 6 months to 6 years, recruited by 143 hospital pediatrics departments in mainland France and French regions overseas (French West Indies and Reunion Island). We used a two-stage random sampling design: in the first stage, the primary sampling units were hospitals and in the second stage, hospitalized children were included. The hospitals were stratified by administrative region and risk of lead poisoning, prevalence of old and substandard housing within the catchment area, and lead-related industrial activity. Hospitals in high-risk areas were intentionally oversampled. Sampling weights were then adjusted by post-stratification based on auxiliary variables (region, gender, age and eligibility for complementary free health insurance (CMUC)) to increase the precision of estimates and make the sample more representative of the population [22]. A questionnaire collecting socioeconomic, behavioral and environmental factors was administered by pediatricians and nurses at the hospitals, along with a blood test. Answers were collected from parents before they received the results of the blood lead test. BLL measurements are described in Etchevers *et al.* 2014 [7].

### 2.2. Statistical Analyses

#### 2.2.1. Sensitivity and Specificity of the Current Lead Risk Questionnaire

First we evaluated the sensitivity and specificity of the current French lead risk screening questionnaire to predict BLLs ≥ 50 µg/L. We considered the questionnaire positive when children met one or more lead screening questionnaire criteria. Two items were not available in the Saturn-Inf questionnaire: parental hobbies involving lead exposure, and child living in, or frequenting, places near an industry emitting lead. Variables were matched as closely as possible with current screening criteria based on guidelines issued by the Direction Générale de la Santé in 2006 [12]. If a parent replied “don’t know” to any question, that child was excluded.

#### 2.2.2. Logistic Regression Modeling

In order to compare the performance of current and new screening criteria, 2 predictive logistic multivariable regression models were constructed from the Saturn-Inf survey sample to predict BLLs ≥ 44 µg/L: one representing current French screening guidelines (current screening criteria model) and a second based on characteristics collected during the 2008–2009 Saturn-Inf national blood lead levels study (new screening criteria model). Since we lacked the power to perform the predictive models on BLLs ≥ 50 µg/L, we chose to implement analysis on the 97.5th percentile of BLLs corresponding to 44 µg/L, in order to increase the number of children over this threshold.

Six out of nine screening criteria were included in the current model. Adult occupation involving lead exposure and child living near industry emitting lead were not available in the Saturn-Inf questionnaire. We did not have enough children with a positive response to the variable “child living with a lead-poisoned (BLL > 100 µg/L) person” to include it in the model.

Twenty-two independent variables for the new screening criteria model were pre-selected a priori from the self-reported data of the Saturn-Inf survey. In addition, a geographic housing risk indicator was created and evaluated. Using a free internet tool based on the national Cadastre map, each child’s address was located in a cadastral section. The Cadastre map represents all the properties built in each municipality in France. A cadastral section is a group of tax parcel units having an average population of 486 people. The risk of living in substandard and old housing was evaluated through a neighborhood housing risk indicator, available at the cadastral section level for municipalities of more than 5000 people and at the municipality level for other cities. This indicator, created by the French National Housing Agency (ANAH) from 2007 tax assessor data, corresponds to the proportion of potentially substandard pre-1949 housing among principal residences in each cadastral section or municipality. Potentially substandard housing is defined as (1) average-quality housing, occupied by a family whose income is below 70% of the poverty line; or (2) low-quality housing in dilapidated condition without toilet, bathroom or central heating, occupied by family with income below 150% of the poverty line. Data were only available for mainland France (*n* = 3625). For 91.5% of children in the sample (3317/3625), addresses were located within a cadastral section but the neighborhood housing risk indicator was available at the cadastral section level for only 38% (1377/3625) of them. The data set was completed with the indicator available at the municipality level. Finally, the neighborhood housing risk indicator was available for 86% of children at the cadastral (38%) or communal (48%) level; 14% were missing.

We performed a backward stepwise selection among the 23 variables and kept the most predictive ones, according to the Nagelkerke *R*^2^, a measure of explained variation calculated on the log likelihood scale [23]. We removed variables one by one until reaching the highest value of Nagelkerke *R*^2^. Interactions in the final multivariable new screening criteria model were examined and considered significant when Nagelkerke *R*^2^ increased. Quantitative variables were introduced as natural spline functions with three degrees of freedom to account for non-linearity, while other variables were introduced linearly. We performed analysis on the complete data set (2445 children) for the mainland Saturn-Inf population sample (*n* = 3625), with no missing data for the included variables. The reported results of the logistic regression models are presented with Odds ratios (OR) and their 95% confidence intervals. For quantitative variables (proportion of substandard housing and occupation rate) OR are expressed for a relative change between the 25th percentile and the 75th, 95th and 99th percentile. These changes may be interpreted as the OR between a moderately low exposure to respectively, moderately high, high and very high exposure. All variables with an OR over 1 were considered as risk factors of elevated BLLs. Strength of the evidence for each variable was evaluated using 95% confidence interval (including 1 or not).

From the final model, we derived area under the curve (AUC) criteria for the receiver operating characteristic (ROC) curves. The AUCs quantify the models’ ability to differentiate individuals having BLL ≥ 44 µg/L and individuals having BLL < 44 µg/L (discrimination). To construct the ROC curves, predicted probabilities of having a BLL ≥ 44 µg/L were calculated for each child using logistic regression equations and sampling weights.

#### 2.2.3. Performance Evaluation of Multivariable Models

The ROC curves were plotted for the current and new screening models and AUCs for the two models were compared, to identify which model performed better. Statistical variance was produced by bootstrap. 500 samples were generated by sampling with replacement of the 2009 Saturn-Inf survey sample. Both predictive models were applied to the bootstrap samples producing 200 ROC curves and associated AUCs for each model.

#### 2.2.4. Internal Validation of Performance of the Better Model

The apparent performance of a model estimated in the population from which it was developed is often overestimated [24,25]. To estimate the internally validated performance of the new model developed in this study, we estimated the optimism of the new model using a bootstrap procedure—which is the most efficient validation technique [26]. The optimism corresponds to the difference between the predictive accuracy on training dataset and validation dataset [24,25]. We generated 200 random samples of 2445 children by drawing samples using replacements from our original sample. A prediction model was fitted to each bootstrap sample (training dataset) using the same criteria for selection (backward based on Nagelkerke *R*^2^) as in the original model and then tested in the original sample (validation dataset). The 200 differences between bootstrap and original sample were averaged to obtain a single estimate of the optimism.

## 3. Results

### 3.1. Sensitivity and Specificity of the Current Lead Risk Questionnaire in Identifying BLLs Higher than 50 µg/L

In the Saturn-Inf sample (*n* = 3831), 23.6% of the children met at least one positive screening criterion, 44.0% no positive criterion and 32.4% of the parents replied “don’t know” to at least one question on the screening questionnaire. Responses from parents to the current risk screening questions are presented in Table 1. Having parents occupationally exposed to lead, or living in pre-1949 housing with renovation work were the most prevalent positive criteria among children with BLLs ≥ 50 µg/L.

**Table 1:** Frequency of positive current lead screening criteria. France, 2008–2009.

Self-Reported Exposure Questions	Observed Responses in Saturn-Inf Sample (*n* = 3831) *	Estimated Prevalence in French Children <7 Years (*N* = 5102,537)	Observed Prevalence of BLLs ≥ 50 µg/L in Saturn-Inf Sample (*n* = 67) *	
	*n*	% (95% CI)	*n*	%
Child recently arrived in France (<1 year)				
No	3802	99.8 (99.6; 99.9)	65	98.5
Yes	10	0.2 (0.1; 0.4)	1	1.5
Child residing with someone who has been lead-poisoned				
No	3333	88.0 (86.0; 89.7)	59	88.1
Yes	3	0.04 (0.01; 0.1)	0	0
Don’t know	436	12.0 (10.3; 13.9)	8	10.9
Adult occupation involves lead exposure				
No	2778	86.8 (84.9; 88.5)	42	82.3
Yes	461	13.2 (11.5; 15.0)	9	17.6
Child drinking tap water where housing has lead pipes				
No	1770	49.4 (45.5; 53.8)	23	34.3
Yes	66	2.2 (1.3; 3.6)	4	6,0
Don’t know	1928	48.4 (44.8; 51.9)	40	59.7
Child living in pre-1949 housing with peeling paint inside				
No	2465	71.8 (65.5; 78.6)	36	57.1
Yes	163	4.8 (3.6; 6.2)	5	7.9
Don’t know	1064	23.4 (20.2; 27.0)	22	34.9
Child living in pre-1949 housing with recent renovation work inside				
No	2379	70.5 (62.6; 79.2)	33	53.2
Yes	290	7.5 (6.1; 9.4)	9	14.5
Don’t know	972	22.0 (19.1; 25.0)	20	32.3
Child living in pre-1949 housing and having tendency to scrape off or nibble paint				
No	2589	75.5 (68.7; 82.6)	36	55.4
Yes	46	0.9 (0.6; 1.5)	5	7.7
Don’t know	1084	23.6 (20.4; 27.3)	24	36.9

* Missing data not presented.

The sensitivity of having BLLs ≥ 50 µg/L where parents reported at least one positive criterion in the screening questionnaire was 0.51 (95% Confidence Interval = (0.26; 0.75)) and specificity was 0.66 (95% CI = (0.62; 0.70)) (Table 2).

**Table 2:** Sensitivity and specificity of the current French lead risk questionnaire in predicting BLLs ≥ 50 µg/L, France 2008–2009.

TP	FP	FN	TN	Missing Data (Don’t Knows and Missing)	Sensitivity (95%CI) *	Specificity (95%CI) *
23	831	17	1402	1558	0.51 (0.26; 0.75)	0.66 (0.62; 0.70)

Abbreviations: TP: true positive; FP: false positive; FN: false negative; TN: true negative; * Estimation based on non-missing data, with sampling weights.

### 3.2. Multivariable Predictive Models

On the basis of current criteria, the multivariable regression model shows that BLLs exceeding 44 µg/L were associated with consumption of tap water in presence of lead pipes, living in pre-1949 housing or housing for which age is unknown and no peeling paint or renovation works inside the dwelling (Table 3).

**Table 3:** Associations between current French screening criteria and BLLs ≥ 44 µg/L (*n* = 2445) in children, France 2008–2009. Results from the logistic multivariable regression model.

Self-Report Exposure Questions		Observed Cases in BLLs ≥ 44 µg/L (*n* = 107)	Estimated Prevalence (%) in BLLs ≥ 44 µg/L	OR	95% CI	
Child recently arrived in France (<1 year)		2	2.3	11.6	0.3	455.5
Child having tendency to scrape off or nibble paint		16	11.6	0.7	0.2	2.7
Adult occupation involves lead exposure		16	7.6	0.6	0.3	1.4
Lead pipes	Type of drinking water					
No	Bottled water	24	29.2	Reference level		
No	Tap water	19	28.1	0.8	0.3	2.0
Yes	Bottled water	2	0.5	0.1	0	1.1
Yes	Tap water	4	6.9	32.6	1.7	626.6
Don’t know	Bottled water	23	12.6	0.3	0.1	1.1
Don’t know	Tap water	35	22.6	1.5	0.3	8.4
Housing age	Peeling paint inside the dwelling					
Built post-1949	No	32	22.1	Reference level		
Built post-1949	Yes	4	4.4	2.9	0.5	18.2
Built pre-1949	No	20	18.4	3.6	1.0	12.2
Built pre-1949	Yes	11	12.0	0.8	0.1	7.2
Don’t know	No	23	36.9	6.2	1.3	29.6
Don’t know	Yes	10	6.3	0.3	0.0	4.7
Housing age	Renovation works inside the dwelling					
Built post-1949	No	27	18.4	Reference level		
Built post-1949	Yes	11	8.5	1.2	0.4	3.7
Built pre-1949	No	18	19.9	3.6	1.0	12.2
Built pre-1949	Yes	13	10.2	0.8	0.2	4.2
Don’t know	No	25	34.2	6.2	1.3	29.6
Don’t know	Yes	8	8.7	0.6	0.1	3.3

The new multivariable screening model has selected 9 criteria: gender (female), child’s recent arrival in France, mother born outside France, parents smoking indoors (more than 5 hours per day), consumption of tap water in presence of lead pipes, living in pre-1949 housing without peeling paint inside, parental occupation involving lead exposure, occupancy rate (≥3 people per room) and proportion of substandard pre-1949 housing per cadastral section or municipality (Table 4). These criteria were positively associated with BLLs exceeding 44 µg/L except for the parental occupation. 95% confidence intervals were large for child’s recent arrival and consumption of tap water with presence of lead pipes probably due to the small sample size. No association was found between BLLs exceeding 44 µg/L and the presence of peeling paint in pre-1949 housing. It may be due to a lack of statistical power. This screening criterion was nonetheless selected in the model and we can notice that there was an interaction between post-1949 housing and the presence of peeling paint with BLLs exceeding 44 µg/L (although not significant).

**Table 4:** Associations between new screening criteria and BLLs ≥ 44 µg/L (*n* = 2445) in children. France 2008–2009. Results from the logistic multivariable regression model.

Variables		Observed Cases for BLLs > 44 µg/L (*n* = 107)	Estimated Prevalence (%) for BLLs > 44 µg/L	OR	95% CI	
Gender of child						
Male		60	47.8	Reference level		
Female		47	52.2	2.4	1.2	5.0
Child recently arrived in France (<1 year)						
No		104	97.7	Reference level		
Yes		2	2.3	6.4	0	1366.7
Mother’s country of birth						
France		73	69.9	Reference level		
Other country		33	30.1	2.9	1.2	6.8
Parents smoke indoors						
No		74	75.7	Reference level		
<1 h/day		13	12.0	3.1	0.9	10.4
1–2 h/day		6	2.6	0.6	0.2	2.2
2–5 h/day		6	2.2	0.1	0	0.6
>5 h/day		7	7.5	8.6	2.7	27.5
Lead pipes	Type of drinking water					
No	Bottled water	24	29.2	Reference level		
No	Tap water	19	28.1	1.0	0.3	3.4
Yes	Bottled water	2	0.5	0.1	0	1.5
Yes	Tap water	4	6.9	16.6	0.2	1321.7
Don’t know	Bottled water	23	12.6	0.2	0.1	0.9
Don’t know	Tap water	35	22.6	1.1	0.2	6.0
Housing age	Peeling paint inside the dwelling					
Built post-1949	No	32	22.1	Reference level		
Built post-1949	Yes	4	4.4	3.1	0.4	25.0
Built pre-1949	No	20	18.4	3.1	1.1	8.5
Built pre-1949	Yes	11	12.0	0.8	0.1	11.0
Don’t know	No	23	36.9	4.8	0.9	25.0
Don’t know	Yes	10	6.3	0.1	0	4.1
Adult occupation involves lead exposure						
No				Reference level		
Yes		16	7.6	0.3	0.1	0.9
Occupancy rate (number of people/number of rooms)						
Change from percentile 25 to percentile 75:0.7 to 1.1				0.6	0.3	1.1
Change from percentile 25 to percentile 95:0.7 to 1.7				1.4	0.8	2.6
Change from percentile 25 to percentile 99:0.7 to 3				3.6	2.0	6.4
Proportion of substandard pre-1949 housing per cadastral section or municipality						
Change from percentile 25 to percentile 75:0.8 to 6.0%				5.0	4.8	5.3
Change from percentile 25 to percentile 95:0.8 to 15.5%				14.2	13.5	15.0
Change from percentile 25 to percentile 99:0.8 to 29.3%				27.7	26.3	29.1

The strongest predictor of BLLs exceeding 44 µg/L was the proportion of substandard pre-1949 housing per geographic area, with an OR of 5 for a change from the 25th percentile to the 75th percentile. The OR was equal to 27.7 for a change from the 25th percentile to the 99th percentile.

### 3.3. Performance of Predictive Models

**Figure 1:**
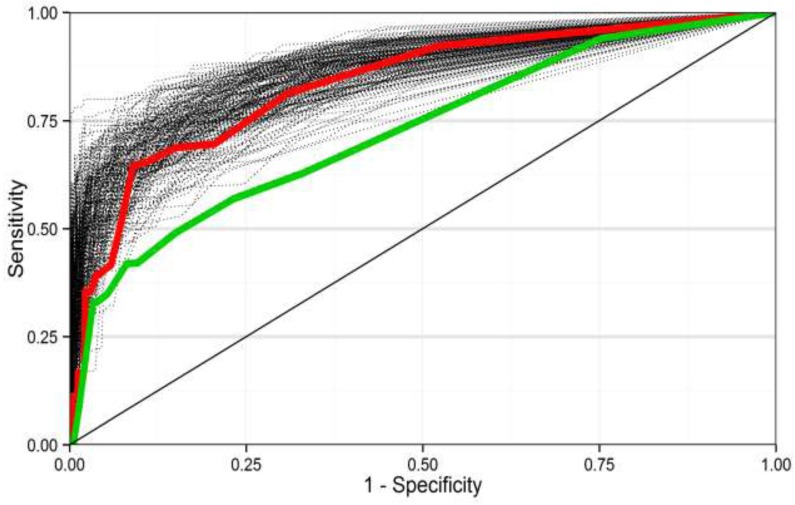
Areas under the receiver operating characteristic curves from the 500 bootstrapped samples for the new screening model (red) and the current screening model (green).

The new screening model, with an AUC of 0.86 (95% CI = (0.80; 0.91)), has a better discrimination ability than the current screening model (AUC = 0.76 (95% CI = (0.69; 0.84))), as shown on Figure 1. The bootstrapped AUC for the new screening model was systematically closer to 1 than the AUC of the current one, meaning it is better able to detect BLLs higher than 44 µg/L. Over-optimism in the AUC value of the new screening criteria model was 0.11.

The new criteria of the new screening model presented higher sensitivity and specificity than the current ones. For lead poisoning screening, we aim to maximize the sensitivity for an acceptable specificity. According to the ROC curve, the highest sensitivity obtained was 0.96 and the corresponding specificity was 0.55.

## 4. Discussion

### 4.1. Sensitivity and Specificity of the Current Lead Risk Questionnaire in Screening BLLs Higher than 50 µg/L

The current lead risk questionnaire performed no better than chance in predicting BLLs ≥ 50 µg/L with a sensitivity of 0.51 (0.26; 0.75) and a specificity of 0.66 (0.62; 0.70). This low precision is due to a small number of children having BLLs ≥ 50 µg/L (67 children) and 39% of missing data or “don’t know” responses in our sample. The “don’t know” responses mainly concerned housing age and lead water pipes. We slightly improved sensitivity (0.65 (0.47; 0.820) by considering “don’t know” responses as positive, but in so doing reduced specificity to 0.44 (0.40; 0.48). These results show that risk factors need to be refined at current lower levels of lead exposure. Many studies in the US have assessed the effectiveness of lead risk questionnaires in detecting children having BLLs ≥ 100 µg/L [27,28,29,30] but none at a level exceeding 50 µg/L. The CDC (Centers for Disease Control and Prevention) lead screening questionnaire published in 1991 had a comparable pooled mean sensitivity and specificity (respectively 0.61 and 0.52) [31]. Better discrimination was obtained by other US questionnaires when they were specifically defined for a dedicated population [31].

### 4.2. Proposal for a New Lead Risk Questionnaire

This study has identified a new combination of individual and geographical lead screening criteria that better targets children having BLLs exceeding 44 µg/L than the guidelines currently used in France. Some current criteria, such as housing age (pre-1949 or unknown), the presence of peeling paint, the presence of lead pipes, the parental occupation (involving lead exposure) and recent arrival of the child in France (<1 year) remain in the new proposal. The new criteria are: gender, mother born outside France, parental smoking indoors, housing occupancy rate and the proportion of substandard pre-1949 housing at cadastral or municipal level.

Being female increases the risk of a BLL higher than 44 µg/L, which is not concordant with most studies, where BLLs are either equivalent for both genders or higher for boys [32,33]. This is probably because some girls with BLLs higher than 44 µg/L have elevated sampling weights in our sample. As a consequence we cannot recommend using gender as a screening criterion.

Having a mother born outside France was identified as a new criterion. This can be a proxy for low socioeconomic status, which is correlated to lead exposure [34,35], and corresponds to lead exposure through traditional remedies, cosmetics or artisanal ceramics [36,37,38,39,40,41,42]. To improve the sensitivity of this criterion (30% risk of having BLLs higher than 44 µg/L), we recommend asking the child’s parents whether they use ceramics, cosmetics or remedies containing lead, as in the US lead risk questionnaires [43]. Immigrant children were also more at risk of having BLLs exceeding 44 µg/L, possibly explained by an environmental lead exposure in their country of origin, as shown by Kaplowitz and coll [44].

We found that child BLLs higher than 44 µg/L were associated with smoking indoors. Other studies in the US have reported an association between cotinine or declared tobacco smoke exposure and BLLs [45,46,47,48].

Living in overcrowded housing (3 or more people per room) is a new criterion. Overcrowding can worsen paint condition and can also be a proxy for poverty. Lanphear *et al.* reported an association between BLLs higher than 100 µg/L and the highest tercile of population density in housing per census block group in New York [49]. In lead risk screening questionnaires in the US, parental rental status, Medicaid eligibility and poverty levels are used to target poor children [27,43].

Residence location can constitute a screening criterion as recommended by the CDC [43]. We found strong associations between BLLs higher than 44 µg/L and the proportion of substandard housing built pre-1949 per cadastral section or municipality with a gradient effect. Krieger *et al.* found similar associations between children with BLLs higher than 100 µg/L and census tracts or zip codes where more than 20% of people live below the poverty line, and more than 27% in pre-1950 housing [18]. Miranda *et al.* reported that year of construction, median income and percentage of African Americans were the best predictors of BLLs at individual tax parcel unit level among children screened in North Carolina [19]. Because many studies in the US have shown that the smaller the geographic level is, the better the prediction of high BLLs [18,50,51], we recommend using the indicator at a cadastral level to achieve better sensitivity. The address can be used as a screening criterion if the child lives in a cadastral section having more than 6% substandard old housing. A web interface could be developed to help medical practitioners to decide, on the basis of the child’s address, whether a blood lead test should be performed. The indicator could also help to identify high-risk cadastral sections that could benefit from remediation and to assess whether future screening programs reach children at high risk, as shown in other studies [20,52,53].

In current French screening guidelines, “don’t know” answers on lead risk questionnaires are ignored. However, we found increased risk associated with “don’t know” answers for housing age and lead pipes exposure. Administering a blood lead test to all children whose parents answer “don’t know” to any question, as in the CDC 1997 lead screening questionnaire [43], must be weighed against the poor resulting specificity. As a consequence, efforts must be made to avoid “don’t know” answers as much as possible.

### 4.3. Limitations

There are some limitations to this study. Assessment of the current lead screening questionnaire, based on only 67 cases with BLLs higher than 50 µg/L, had high uncertainty and did not include all questions (2 missing). Our use of sampling weights and post-stratification do however add accuracy to our estimations.

With regard to the new screening criteria, we aimed to perform the analysis for BLLs higher than 50 µg/L but had sufficient statistical power only for BLLs higher than 44 µg/L. It is possible that some variables kept in our model might not have been selected in a model based on BLLs greater than 50 µg/L. Even for BLLs higher than 44 µg/L, our estimates for the “recent arrival of the child in France” and “consumption of tap water with presence of lead pipes” had a high level of uncertainty because of the small number of children concerned.

The interaction between peeling paint and age of housing was positive (though not significant) only for housing built post-1949 (the year in which white lead-based paint ceased to be used in France). This may be due to a misclassification of housing age by parents. The number of children living in housing with peeling paint was insufficient to study the association accurately. We suggest keeping each of these criteria separate, in order to improve sensitivity in predicting BLLs higher than 44 µg/L. In the Saturn-Inf questionnaire, we asked whether the child’s housing was built pre- or post-1949. However, the presence of lead-based paint in homes built between 1949 and 1993 reported in France showed lead concentration at ≥1 mg/cm^2^ in 32% of homes built pre-1961 and 18% of homes built pre-1974—*versus* 50% for homes built pre-1949 [10,54]. Further studies are needed to refine the construction date question.

We used an ecological indicator at two spatial scales—municipality and cadastral section—having a heterogeneous population size. This has probably reduced the accuracy of the indicator. Moreover, the housing data collected in 1970 are infrequently updated, probably resulting in an overestimation of the number of old and potentially substandard dwellings as well as an underestimation of the association with BLLs.

To adjust for the over-optimistic performance of the 2009 Saturn-Inf model, we performed an internal validation using a bootstrapping procedure on 200 bootstrap data sets generated from the original sample. Estimated over-optimism was 0.11. However, we did not have another population on which to perform an external validation, as recommended by many authors [24,25].

Our new proposal focused only on child exposure in the home. However a new questionnaire should include questions about well-identified risk factors such as the child visiting other places built pre-1949, the child visiting or living near an industry emitting (or having emitted) lead, parental hobbies involving exposure to lead and the child having a sibling or playmate having (or having had) lead poisoning.

## 5. Conclusions

This study has shown that the current lead risk screening criteria for BLL higher than 100 µg/L needs to be improved to detect children with BLLs higher than 50 µg/L. We identified a combination of 8 screening criteria that could better target children with BLLs higher than 44 µg/L. In addition to the individual self-reported risk factor, we propose an ecological indicator of old and substandard housing, available nationwide, to help medical practitioners to improve the screening rate of children at risk, and local health departments to target geographic areas where primary prevention activities can be implemented before children are exposed.
